# Anti-Hyperuricemic and Nephroprotective Effects of Hydrolysate Derived from Silkworm Pupae (*Bombyx mori*): In Vitro and In Vivo Study

**DOI:** 10.3390/nu17091596

**Published:** 2025-05-06

**Authors:** Yuting Fan, Zhencong Yang, Xiao Lin, Zhoujin Xu, Lixia Mu, Qingrong Li, Xuli Wu

**Affiliations:** 1 School of Public Health, Health Science Center, Shenzhen University, Shenzhen 518060, China; fanyuting@szu.edu.cn (Y.F.); 2310246004@email.szu.edu.cn (Z.Y.); 2070245003@email.szu.edu.cn (X.L.); 2310246025@email.szu.edu.cn (Z.X.); 2 Sericulture and Agro-Processing Research Institute, Guangdong Academy of Agricultural Sciences, Guangzhou 510640, China; lisa1980119@126.com (L.M.); liqingrong@gdaas.cn (Q.L.)

**Keywords:** silkworm pupae, peptides, anti-hyperuricemic, anti-inflammatory, xanthine oxidase

## Abstract

Background: Hyperuricemia is a prevalent metabolic disorder characterized by elevated serum uric acid (UA) levels. Methods: In this study, hydrolysate (SPP) derived from silkworm pupae protein was isolated and identified, demonstrating anti-hyperuricemic activity. The research aimed to investigate its anti-hyperuricemic and nephroprotective effects, along with potential mechanisms, through in vitro assays and in vivo experiments using potassium oxonate/hypoxanthine-induced hyperuricemic mice. Results: The SPP exhibited significant xanthine oxidase (XOD) inhibitory activity, with an IC_50_ value of 7.41 mg/mL. Furthermore, SPP administration effectively reduced serum UA, blood urea nitrogen (BUN), creatinine levels, and renal pro-inflammatory cytokines in hyperuricemic mice. Mechanistic studies revealed that the anti-hyperuricemic effects of SPP may involve XOD inhibition and the modulation of renal UA transporters, specifically upregulating organic anion transporter 1 (OAT1) and ATP-binding cassette subfamily G member 2 (ABCG2) expression. Histopathological analysis and inflammatory cytokine profiling further demonstrated that SPP alleviated renal inflammation and pathological damage. Conclusions: These findings suggest that SPP possesses a notable urate-lowering efficacy and renal protective properties, highlighting its potential as a therapeutic agent for the management and prevention of hyperuricemia (HUA).

## 1. Introduction

Xanthine oxidase (XOD), a rate-limiting enzyme in uric acid (UA) biosynthesis, catalyzes the sequential oxidation of hypoxanthine to xanthine and, subsequently, xanthine to UA during purine metabolism [1,2]. This dual catalytic role establishes XOD as a critical therapeutic target for hyperuricemia (HUA) management [3,4,5,6]. Current clinical interventions primarily employ XOD inhibitors such as allopurinol and febuxostat, while complementary strategies focus on enhancing UA excretion (e.g., benzbromarone and probenecid) [7,8] or accelerating UA catabolism (e.g., rasburicase) [9]. However, clinical trials have revealed significant adverse effects associated with these agents, including nephrotoxicity [10], drug-induced nephropathy [11], and hypersensitivity reactions [12]. These safety concerns underscore the urgent need to develop natural alternatives with improved safety profiles, enhanced efficacy, and cost-effectiveness. Emerging evidence suggests that bioactive macromolecules may offer promising therapeutic avenues. For instance, fucose-rich polysaccharides have demonstrated UA-lowering effects through the modulation of renal urate transporters, including glucose transporter 9 (GLUT9), ATP-binding cassette subfamily G member 2 (ABCG2), organic anion transporter 1 (OAT1), and urate anion transporter 1 (URAT1) [13]. Beyond polysaccharides, other biocompatible biomacromolecules such as proteins and unsaturated fatty acids are gaining research attention due to their favorable biosafety profiles and minimal side effects [9,14].

Bioactive protein hydrolysates and peptides have emerged as promising therapeutic agents in nutritional interventions, exhibiting diverse therapeutic properties, including anti-hyperuricemic [15], anti-inflammatory [16], and gastroprotective activities [17]. The regulation of serum uric acid levels has gained increasing clinical attention due to its established association with multiple pathological conditions, including gout pathogenesis [18], cardiovascular complications [19], and chronic kidney disease progression [20]. Emerging evidence further suggests that sustained hyperuricemia exacerbates metabolic syndrome through inflammatory cascades and oxidative stress pathways [21]. Notably, a bioactive peptide derived from aqueous extracts of *Oryza sativa* fruit pericarps demonstrated significant urate-lowering effects in hyperuricemic murine models through the following multi-target mechanisms: the inhibition of XOD activity, the downregulation of urate transporter 1 (URAT1) and NLRP3 inflammasome expression, and the suppression of pro-inflammatory mediators [22].

Furthermore, novel XOD-inhibitory peptides isolated from marine sources (*Litopenaeus vannamei* and *Portunus trituberculatus*) exhibited therapeutic efficacy in HK-2 renal tubular epithelial cell models of hyperuricemia, achieving urate reduction via the dual mechanisms of suppressing proinflammatory cytokine production and modulating uric acid transporter expression. Molecular docking analyses revealed that key intermolecular interactions—particularly hydrogen bonding, salt bridge formation, and electrostatic forces—critically stabilized the binding conformations between these peptides and the XOD active site [15]. Accumulating preclinical evidence substantiates the potential of bioactive protein derivatives as adjuvant therapies for hyperuricemia management, though clinical validation remains necessary to establish translational relevance.

Silkworm (*Bombyx mori*) represents an economically significant edible insect species with dual biological and commercial value. In several Asian nations, particularly China and India, silkworm derivatives serve as crucial sources of animal protein. The sericulture industry annually generates substantial quantities of by-products, including larvae, pupae, and frass, during silkworm cultivation processes [23]. Silkworm pupae protein contains a nutritionally balanced composition rich in bioactive components, demonstrating considerable potential for industrial applications [24]. Notably, SPP constitutes a key ingredient in traditional Chinese medicine formulations prescribed for metabolic disorders such as hypertension and hepatic steatosis [25]. Remarkably, essential amino acids comprise over 50% of the total amino acid profile in SPP [23]. Cumulative research has validated the multifunctional pharmacological properties of SPP hydrolysates, including myostimulatory effects [26], immunomodulatory enhancement [27], antioxidant capacity [28], and antitumor activity [29]. Despite these advancements, research investigating the anti-hyperuricemic potential and associated molecular mechanisms of SPP remains in its preliminary stages.

This study systematically investigated the anti-hyperuricemic potential of enzymatically prepared silkworm pupae protein hydrolysate (SPP). The research encompassed the preparation, isolation, and structural characterization of SPP, followed by a comprehensive evaluation of its therapeutic effects through integrated in vitro and in vivo approaches. XOD inhibitory activity was quantitatively assessed using in vitro enzymatic assays. Subsequent in vivo investigations evaluated SPP’s capacity to modulate serum UA levels, renal functional markers (blood urea nitrogen and creatinine), and inflammatory responses in a hyperuricemic murine model. Mechanistic studies further elucidated the regulatory effects of SPP on renal UA transporters to delineate its urate-lowering pathways. These findings will not only advance the utilization of silkworm pupae protein for industrial-scale bioactive compound production, but also establish a theoretical framework for developing functional food components targeting HUA prevention.

## 2. Materials and Methods

### 2.1. Chemicals and Materials

XOD (25 U/mL), xanthine, and potassium oxonate were purchased from Sigma-Aldrich Chemical Co. (St. Louis, MO, USA). Hypoxanthine was acquired from Macklin Biochemical Technology Co., Ltd. (Shanghai, China). Primary antibodies against ABCG2 and β-actin were procured from Cell Signaling Technology (Danvers, MA, USA). Antibodies targeting OAT1, NLRP3, Caspase-1, and IL-1β were obtained from Affinity Biosciences (Jiangsu, China). Horseradish peroxidase (HRP)-conjugated goat anti-mouse IgG secondary antibody was supplied by ZSGB-BIO (Beijing, China). Enhanced chemiluminescence (ECL) detection reagents were sourced from Bio-Rad Laboratories (Hercules, CA, USA). All other chemicals were of analytical reagent grade or higher purity.

### 2.2. Preparation and Enzymatic Hydrolysis of Silkworm Pupae Protein

*Bombyx mori* (P50 strain) larvae were provided by the Guangdong Academy of Agricultural Sciences (Guangzhou, China) and reared under standardized laboratory conditions. Silkworm pupae protein extraction followed a previously established protocol [30]. Briefly, pupae were cryogenically pulverized in liquid nitrogen and defatted using ethyl acetate. The resultant powder was suspended in deionized water (1:10 *w*/*v*), alkalized to pH 10.0 with 1 M NaOH, and centrifuged (4 °C, 5000× *g*, 20 min). The pellet was acidified to pH 4.0 with 1 M HCl, incubated for 4 h at 4 °C, and centrifuged to isolate the precipitated protein.

Enzymatic hydrolysis was optimized via response surface methodology (RSM) using Design-Expert^®^ software 6.0.1. A central composite design (CCD) was implemented with XOD inhibitory activity as the response variable, incorporating the following four independent parameters: temperature (A), pH (B), enzyme-to-substrate ratio (C), and hydrolysis duration (D). The protein solution was preconditioned to optimal parameters (Table S1) before bromelain supplementation (EC 3.4.22.32, Sigma-Aldrich, St. Louis, MO, USA). Enzymatic activity was terminated by thermal inactivation (95 °C, 10 min), followed by centrifugation (8000× *g*, 15 min), to collect the hydrolysate supernatant, which was lyophilized for subsequent analyses.

### 2.3. Molecular Weight and Peptide Sequence Analysis of SPP

The molecular weight and peptide sequence of SPP were analyzed following our previous methodology [31]. Online chromatographic separation was performed using an Easy nLC 1200 system (Thermo Fisher Scientific, Waltham, MA, USA) for molecular weight determination. Peptide sequencing was carried out with a Q Exactive mass spectrometer (Thermo Fisher Scientific, MA, USA) equipped with a Nano Flex ion source. The acquired spectra were processed through PEAKS Studio 8.5 software for sequence matching against theoretical spectra retrieved from the UniProtKB protein database.

### 2.4. Assessment of XOD Inhibitory Activity

The XOD inhibitory activity was evaluated through an optimized UA formation assay adapted from established protocols [32]. The reaction mixture, comprising 30 μL of XOD (0.1 U/mL) and test compounds, underwent pre-incubation at 37 °C for 5 min. Enzymatic reactions were initiated by adding 60 μL of xanthine substrate (150 μM), with baseline absorbance recorded immediately using a BioTek Synergy H1 microplate reader (BioTek Instruments, Winooski, VT, USA). Subsequent kinetic measurements at 295 nm were conducted under controlled conditions (25 °C) for 25 min before reaction termination. PBS substitution for test samples was used for blank control. PBS replacement of the XOD enzyme was used for sample control. Inhibition efficiency was quantified using the following relationship:

Inhibition rate% = 1 − (A_3_ − A_4_)/A_2_ − A_1_ × 100%

The experimental groups were defined as follows: A_1_—blank control group; A_2_—complete reaction group; A_3_—test compound reaction group; and A_4_—compound control group. The IC_50_ obtained from the inhibition curve represents the concentration of an inhibitor that inhibits 50% of XOD activity [33].

### 2.5. Animal Experiments

All experimental procedures were conducted in compliance with the National Institutes of Health Guidelines (China) and approved by the Institutional Animal Care and Use Committee of Shenzhen University Medical School (Approval No.: IACUC-202300134). Male ICR mice (4 weeks old, 26 ± 2 g) were obtained from the Guangdong Medical Laboratory Animal Center (Foshan, China). Following a 7-day acclimatization period under controlled conditions (23 ± 1 °C, 12 h light/dark cycle) with ad libitum access to food and water, the animals were randomly allocated into the following six experimental groups (n = 7/group): normal control (NC), model control (MC), febuxostat (FEB, 8 mg/kg/d), and three SPP dose groups (SPP-L: 0.25 g/kg/d, SPP-M: 0.5 g/kg/d, and SPP-H: 1 g/kg/d).

The hyperuricemia model was established through daily intraperitoneal injections of potassium oxonate (PO, 240 mg/kg) and hypoxanthine (120 mg/kg) suspended in 0.5% CMC-Na for 7 days. While the NC and MC groups received the vehicle (0.5% CMC-Na), therapeutic interventions commenced on day 8, with drug administration via oral gavage 1 h post-PO injection for 21 consecutive days. Biological samples were collected on day 28 following CO_2_ euthanasia. Serum was isolated through centrifugation (3000× *g*, 20 min, 25 °C) and stored at −80 °C alongside snap-frozen hepatic/renal tissues. Selected kidney specimens were fixed in 4% paraformaldehyde (PFA) for histological analysis. The complete treatment protocols are detailed in Supplementary Table S2.

### 2.6. Biochemical Indexes Analysis

Serum UA quantification was performed using enzymatic colorimetry with commercial assay kits (Jiancheng Bioengineering Institute, Nanjing, China), according to the manufacturer’s protocols. Parallel biochemical analyses determined blood urea nitrogen (BUN), serum creatinine levels, and XOD activity, employing identical kit specifications under standardized conditions (37 °C, wavelength-specific detection).

### 2.7. Detection of Inflammatory Cytokine Levels in Kidney

Renal cytokine profiling was conducted through standardized tissue processing protocols. Briefly, kidney specimens were pulverized in liquid nitrogen, followed by homogenization in ice-cold phosphate buffer (pH 7.4). The resultant homogenates underwent centrifugation (12,000× *g*, 10 min, 4 °C) to obtain clarified lysates. Pro-inflammatory mediators, including tumor necrosis factor-α (TNF-α), interleukin-6 (IL-6), and interleukin-1β (IL-1β) concentrations, were determined using species-specific ELISA kits (Shanghai Jianglai Biotechnology Co., Ltd., Shanghai, China) following the manufacturer’s protocols, with optical density measurements normalized against total protein content.

### 2.8. Histological Analysis

Histopathological evaluation was performed using standardized protocols. Renal specimens were immersion-fixed in 4% paraformaldehyde (PFA) for 48 h, followed by gradient ethanol dehydration and paraffin embedding. Tissue sections (5 μm thickness) were prepared using a rotary microtome, mounted on poly-L-lysine-coated slides, and stained with hematoxylin-eosin (H&E). Histoarchitecture was examined under a Nikon Eclipse Ti-SR inverted microscope (Nikon Instruments Inc., Tokyo, Japan) with 200× magnification using NIS-Elements imaging software (v4.6), with a particular focus on glomerular morphology and tubular integrity.

### 2.9. Western Blot Analysis

Protein expression profiling was conducted through standardized Western blotting protocols according to established methodology [34]. Renal tissue lysates were prepared using RIPA buffer, with protein concentrations determined by BCA assay. Aliquots containing 30 μg of protein were resolved on 10% SDS-polyacrylamide gels and transferred onto PVDF membranes (0.45 μm, Millipore, Burlington, MA, USA) using semi-dry electrophoretic transfer. The membranes were blocked with 5% non-fat milk in TBST for 2 h at room temperature before incubation with primary antibodies against urate transporters (ABCG2, [1:1000], OAT1 [1:800]), inflammasome components (NLRP3 [1:1000], Caspase-1 [1:1200]), and inflammatory mediators (IL-1β [1:1500]), with β-actin (1:5000) serving as a loading control (all antibodies from Abcam, Cambridge, UK). Following overnight incubation at 4 °C, the membranes were probed with HRP-conjugated secondary antibodies (1:5000) for 1 h at 25 °C. Immunoreactive bands were visualized using SuperSignal™ West Pico PLUS Chemiluminescent Substrate (Thermo Fisher Scientific) with 5 min of exposure on a ChemiDoc™ MP Imaging System (Bio-Rad Laboratories, Hercules, CA, USA). Densitometric analysis was performed using ImageJ software 1.51kwith normalization to β-actin expression.

### 2.10. qRT-PCR Analysis

Total RNA was extracted from kidney tissue and reverse-transcribed using the PrimeScript RT Master Mix kit (Takara Biomedical, Beijing, China)following the manufacturer’s instructions. Quantitative real-time PCR (qPCR) was performed on a qTOWER3 thermal cycler (Analytik-Jena, Jena, Germany). Relative gene expression levels were normalized against β-actin expression and analyzed using the 2^−ΔΔCt^ method [35]. Primer sequences are provided in Table S3.

### 2.11. Statistical Analysis

All experiments were performed in triplicate. Data are expressed as mean ± SD. Statistical analyses and figure arrangements were conducted using GraphPad Prism 7.0 software (GraphPad Software Inc., San Diego, CA, USA), with differences determined by one-way ANOVA followed by Duncan’s test. The labels * *p* ≤ 0.05, ** *p* ≤ 0.01, *** *p* ≤ 0.001, and **** *p* ≤ 0.0001 represent annotations of statistical significance.

## 3. Results

### 3.1. Preparation of SPP

Response surface methodology was employed to optimize the enzymatic hydrolysis process to establish the optimal preparation conditions. The experimental data were subjected to variance analysis using the Design-Expert 8 software, with the results detailed in Table S4. The regression model demonstrated a significant validity (*p* < 0.05), while the lack-of-fit term showed no significance (*p* = 0.1311 > 0.05), confirming the model’s suitability for process prediction and analysis. Based on F-values, the factors influencing the response value were ranked as A > D > B > C. Three-dimensional response surface plots and two-dimensional contour plots (Figure S1) revealed the interactions between the variables and their effects on the response values. Through comprehensive analysis, the optimal hydrolysis parameters were determined as follows: a reaction temperature of 55 °C, pH of 7.0, duration of 5 h, and enzyme-substrate ratio of 4000 U/g, which maximized XOD inhibitory activity.

### 3.2. Molecular Weight and Peptide Sequence of SPP

The molecular weight distribution and peptide composition of SPP were characterized using LC-MS/MS analysis coupled with PEAKS Studio 8.5 bioinformatics processing under optimized hydrolysis conditions.

The enzymatic hydrolysis of SPP generated 1,130 peptide fragments (300–4000 Da), with predominant molecular weight distributions at <1000 Da (39.20%) and 1000–2000 Da (52.92%). Only nine peptides (0.80%) exhibited higher molecular weights (3000–4000 Da), as shown in Figure S2. Notably, previously reported XOD inhibitory peptides, including AEAQMWR [15], ALPM [36], PEW [36], and MAIGLW [37], all fall within the <1000 Da range, consistent with observed bioactivity patterns. The ten most abundant peptides (Table 1) were enriched in hydrophobic residues (Leu, Ile, Val, and Pro) and aromatic amino acids (Phe and Tyr), with asparagine (Asn) also being prominently represented. This amino acid profile suggests potential XOD inhibition mechanisms mediated by hydrophobic interactions within the enzyme’s catalytic pockets [38]. The combined evidence, including 82.12% of peptides being below 2000 Da and the characteristic amino acid composition, strongly supports the anti-hyperuricemic potential of SPP.

### 3.3. XOD Inhibitory Activity

**Figure 1:**
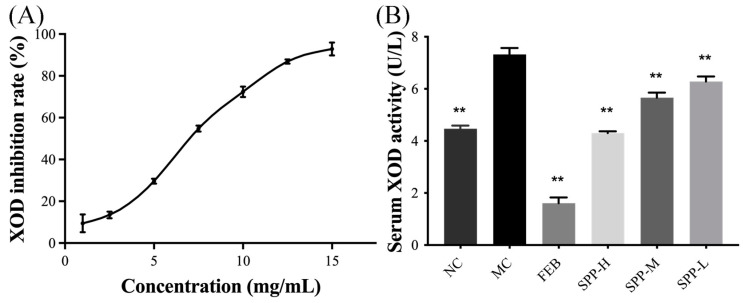
Effect of SPP on XOD activity in vitro and in vivo. (**A**) XOD inhibitory rate in vitro and (**B**) serum XOD activity. ** *p* < 0.01 versus the MC group.

XOD, the rate-limiting enzyme in UA biosynthesis, serves as a critical biomarker for evaluating anti-hyperuricemic agents both in vitro and in vivo. In vitro analysis revealed significant XOD inhibitory activity of SPP, with an IC_50_ of 7.41 mg/mL (Figure 1A), demonstrating a superior efficacy compared to oyster protein hydrolysate (IC_50_ 60.12 ± 1.34 mg/mL) [37], though slightly less potent than that of yellowfin tuna hydrolysate (IC_50_ 2.50 mg/mL) [39]. In vivo evaluation (Figure 1B) showed dose-dependent XOD inhibition, with high-dose SPP (1 g/kg BW) achieving 41.24% suppression, exceeding normal control levels, compared to febuxostat’s 8 mg/kg BW efficacy. The significant suppression of serum XOD activity was observed across treatment groups (SPP-H: 41.24%, SPP-M: 22.60%, and SPP-L: 14.13%) versus the model control (*p* < 0.01). This dose-responsive inhibition pattern confirms SPP’s mechanism of UA reduction through XOD activity modulation. The XOD activity of the SPP-H group was even lower than that of the NC group, indicating that SPP could effectively inhibit the production of UA by suppressing XOD activity.

### 3.4. SPP Alleviated Kidney Damage in Hyperuricemic Mice

**Figure 2:**
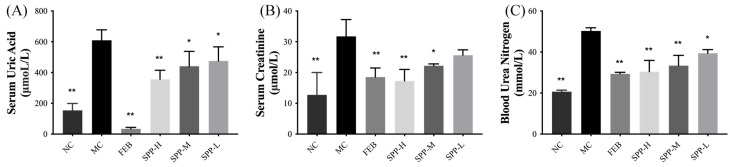
In vivo anti-hyperuricemic activity of SPPs. (**A**) Serum uric acid, (**B**) serum creatinine, and (**C**) blood urea nitrogen. Values are expressed as mean ± SD. The significant difference is shown at * *p* < 0.05, ** *p* < 0.01 versus the MC group.

The therapeutic potential of SPP regarding urate metabolism was evaluated in hyperuricemic ICR mice. Successful establishment of the hyperuricemia model was confirmed by the markedly elevated serum UA levels (609.8 ± 67.65 μM) in the MC group after 7 days of PO administration. As shown in Figure 2A, both febuxostat and SPP interventions induced significant reductions in serum UA levels (*p* < 0.05), demonstrating SPP’s systemic urate-lowering efficacy in vivo. Renal functional biomarkers, including blood urea nitrogen (BUN) and serum creatinine (SCr), were analyzed to assess kidney impairment (Figure 2B,C). The hyperuricemic groups exhibited significantly elevated SCr levels, which were dose-dependently attenuated by SPP treatment (0.50–1.00 g/kg/d). Notably, the high-dose SPP group (SPP-H) showed superior SCr reduction compared to the febuxostat-treated group (*p* < 0.01). Parallel improvements were observed in BUN levels (Figure 2C), with the SPP treatment groups displaying significant decreases relative to the MC group (*p* < 0.05). These coordinated reductions in SCr and BUN, established clinical markers of renal dysfunction [40], suggest SPP’s capacity to mitigate hyperuricemia-induced nephrotoxicity. The observed biomarker normalization implies the potential restoration of renal filtration function, highlighting SPP’s dual therapeutic action on both urate homeostasis and kidney protection.

### 3.5. Histopathological Examination

**Figure 3:**
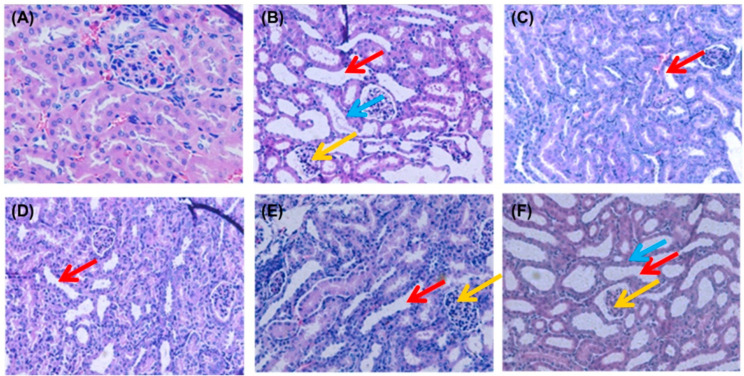
Effect of SPP on the histopathology of kidney tissues: (**A**) NC group; (**B**) MC group; (**C**) FEB group; (**D**) SPP-H group; (**E**) SPP-M group; and (**F**) SPP-L group. Original magnification, ×200 (**A**–**F**); blue arrow: exfoliated epithelioid cells; red arrow: dilated kidney tubules; yellow arrow: interstitial cellular infiltration.

HUA directly induces renal structural damage and functional impairment. To further confirm the biochemical findings, kidney histopathology was analyzed via H&E staining (Figure 3). The NC group exhibited intact glomerular capillaries and well-defined renal tubules with no inflammatory infiltrates. In contrast, PO-induced hyperuricemic mice (MC group) displayed severe nephropathic alterations, including the detachment of tubular epithelial cells (blue arrows), tubular lumen dilation (red arrows), and diffuse inflammatory cell infiltration in interstitial regions (yellow arrows). These pathological features align with established HUA-induced nephropathy models [36]. Remarkably, both the SPP- and febuxostat-treated groups showed the marked attenuation of these abnormalities. Glomerular architecture and tubular integrity were preserved, with minimal cellular swelling and degenerative changes. SPP administration dose-dependently reduced tubular dilation and inflammatory infiltration severity. This histopathological improvement, consistent with serum biomarker normalization (Figure 2), collectively demonstrates SPP’s capacity to alleviate HUA-induced nephropathy by restoring the renal microstructure and mitigating inflammation. Although our analysis provided detailed assessment of tubular epithelial injury and interstitial fibrotic remodeling, the histological evaluation did not encompass peritubular microvascular architecture or glomerular compartment analysis. Besides, the current findings, while demonstrating qualitative nephropathic alterations, were limited by a lack of quantitative enumeration-a methodological constraint requiring future resolution through stereological analysis.

### 3.6. Nephrotic Inflammation in Mice

**Figure 4:**
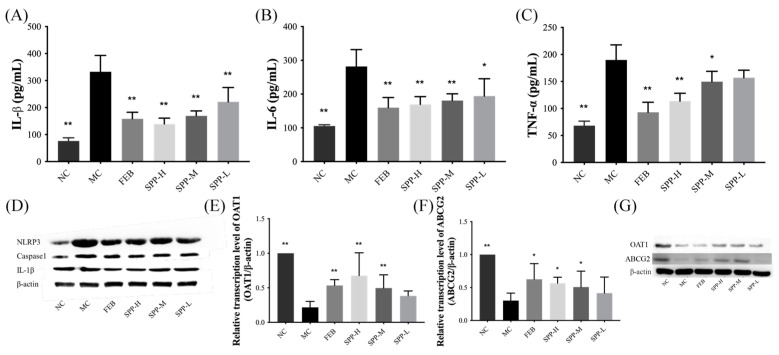
The levels of IL-1β, IL-6, TNF-α, OAT1, and ABCG2 in kidneys. (**A**) IL-1β; (**B**); IL-6; (**C**) TNF-α; (**D**) Western blot analysis of IL-1β, caspase-1, and NLRP3; (**E**) OAT1; (**F**) ABCG2; and (**G**) Western blot analysis of OAT1 and ABCG2.Values are expressed as mean ± SD. The protein levels were normalized to those of β-actin. The significant difference is shown at * *p* < 0.05, ** *p* < 0.01 versus the MC group.

Systemic inflammation is recognized as a pathological hallmark of HUA [41]. As shown in Figure 4A–D, we quantified the pro-inflammatory cytokines (TNF-α, IL-6, and IL-1β) in renal tissues using ELISA. Compared to the NC group, the MC group displayed significantly elevated levels of TNF-α, IL-1β, and IL-6 (*p* < 0.01), indicating an enhanced inflammatory response. However, treatment with febuxostat and SPP reduced these pro-inflammatory factors to varying degrees, with the FEB and SPP-H groups demonstrating particularly strong inhibitory effects. Notably, the high-dose SPP group showed a trend toward a superior suppression of IL-1β compared to febuxostat (Figure 4A), although this difference did not reach statistical significance.

To further characterize inflammatory responses, we analyzed the protein expression levels of NLRP3, caspase-1, and IL-1β via Western blotting. The renal expression of these markers was markedly upregulated in the MC group. As illustrated in Figure 4D, both febuxostat and SPP treatment modestly downregulated these proteins, though no statistically significant differences were observed between groups. These findings suggest that SPP effectively mitigates HUA-induced systemic inflammation, potentially through NLRP3 pathway inhibition.

### 3.7. Expression of UA Transporter Proteins

ATP-binding cassette subfamily G member 2 (ABCG2) and organic anion transporter 1 (OAT1) play pivotal roles in renal uric acid excretion. OAT1 mediates UA uptake into proximal tubular epithelial cells and other organic anions from the bloodstream into tubular cells for subsequent excretion [42]. ABCG2, positioned on the apical membrane of renal and intestinal epithelial cells, facilitates UA anion secretion [42]. As demonstrated in Figure 4E–G, qRT-PCR and Western blot analyses revealed significant downregulation in both the mRNA and protein expression levels of OAT1 and ABCG2 in the MC group. Notably, therapeutic intervention with febuxostat and SPP effectively counteracted this suppression. The high-dose SPP treatment restored transporter expression levels to near-normal values, achieving an efficacy comparable to the FEB group. These findings suggest that SPP enhances UA excretion capacity through the transcriptional and post-translational regulation of key renal transporters, aligning with previous reports on protein hydrolysates ameliorating hyperuricemia [43].

### 3.8. Short-Chain Fatty Acid Profiles in Hyperuricemic Mice

**Figure 5:**
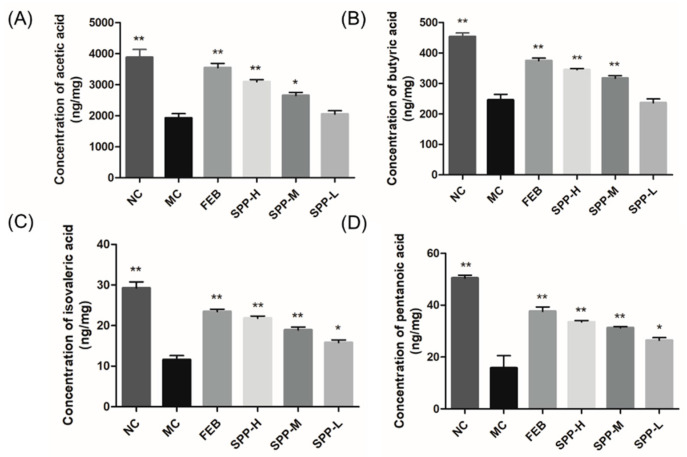
Concertation of SCFAs in hyperuricemic mice. (**A**) Acetic acid; (**B**) butyric acid; (**C**) isovaleric acid; and (**D**) pentanoic acid. The significant difference is shown at * *p* < 0.05, ** *p* < 0.01 versus the MC group.

Emerging evidence indicates that short-chain fatty acids (SCFAs) modulate hyperuricemia through multiple mechanisms, including serving as anti-inflammatory mediators to attenuate gout pathogenesis and fueling intestinal epithelial metabolism to facilitate UA excretion [44]. Four major SCFAs, including acetic acid, butyric acid, isovaleric acid, and pentanoic acid, were quantitatively assessed (Figure 5). Comparative analysis revealed markedly reduced concentrations of all detected SCFAs in the MC group compared to the NC controls (*p* < 0.01). SPP supplementation dose-dependently restored fecal SCFA content, though statistical significance was not achieved in the low-dose group. Notably, febuxostat administration similarly enhanced SCFA production, contrasting with the xanthine oxidase inhibitor allopurinol [43], which exhibited no such regulatory capacity. This differential pharmacodynamic profile highlights a unique mechanism distinguishing SPP and febuxostat from conventional urate-lowering therapies.

## 4. Discussion

Silkworm pupae protein exhibits an optimal amino acid profile complemented by bioactive peptides and phenolic compounds, showing significant promise in functional food development and nutraceutical production. Pharmacological studies validate its role as a therapeutic constituent in hepatoprotective formulations, particularly for managing glucose dysregulation and dyslipidemia-associated pathologies.

In this study, hydrolysate derived from silkworm pupae protein was isolated and identified, demonstrating anti-hyperuricemic activity. The hydrolysate exhibited no observable adverse effects under the tested conditions. These preliminary findings suggest an acceptable biocompatibility, though extended in vivo evaluations remain necessary to fully establish safety profiles. The present study systematically demonstrated that SPP exerts multi-target therapeutic effects against hyperuricemia through XOD inhibition and renal transporter modulation. SPP directly inhibited XOD, the rate-limiting enzyme in urate biosynthesis, thereby reducing uric acid production. Concurrently, it restored renal uric acid excretion capacity by transcriptionally regulating the ABCG2 and OAT1 transporters. Additionally, SPP mitigated systemic inflammation through NLRP3 inflammasome suppression while elevating SCFAs levels. Notably, SPP exhibited superior XOD inhibitory activity (IC_50_ 7.41 mg/mL), potentially attributable to its unique peptide profile dominated by hydrophobic and aromatic residues (<2000 Da). These structural characteristics likely enhanced interactions with XOD’s catalytic pockets, as hydrophobic forces are critical for enzyme-inhibitor binding [38]. The dose-dependent suppression of serum XOD activity (41.24% at 1 g/kg BW) corroborates findings from tuna hydrolysates [39], yet SPP demonstrated additional renal protective effects absent in previous models. This dual functionality, simultaneously reducing UA production and mitigating nephrotoxicity, positions SPP as a comprehensive therapeutic agent surpassing conventional XOD inhibitors like allopurinol. The restoration of ABCG2/OAT1 expression by high-dose SPP administration (comparable to febuxostat) provides mechanistic insights into its uricosuric effects. While previous studies established OAT1’s role in UA reabsorption [42], our work further demonstrates that peptide hydrolysates can transcriptionally regulate these transporters, potentially through nutrient-sensing pathways in renal epithelia. Equally significant is SPP’s ability to modulate gut-derived SCFAs while suppressing renal NLRP3 inflammasome activation. The observed correlation between fecal SCFA restoration and attenuated IL-1β levels (Figure 4A and Figure 5) supports emerging paradigms of gut–kidney axis regulation in HUA [44]. Unlike allopurinol, which solely targets XOD, SPP’s concurrent elevation of butyrate, a known NLRP3 inhibitor, may synergistically disrupt the inflammatory cascade driving gout progression. This dual anti-inflammatory mechanism (systemic and intestinal) could explain SPP’s superior nephroprotection compared to febuxostat in histopathological assessments.

## 5. Conclusions

In conclusion, SPP represents a promising natural alternative for HUA management, integrating enzymatic inhibition, transporter regulation, and microbiome modulation, a therapeutic triad unattainable by current pharmacotherapies. Our findings underscore the potential of insect-derived hydrolysates as functional food ingredients for metabolic disorder intervention.

## Figures and Tables

**Table 1:** The most abundant 10 peptides in SPP.

Peptide	Mass	Length	Peptide Score	Relative Intensity (%)	Area
YHFGVPVG	874.4337	8	875.4418	12.39	11,100,000
FITPF	623.3318	5	624.3403	16.80	7,740,000
FLTPF	623.3318	5	624.3403	16.80	7,740,000
WFITPF	809.4112	6	810.4222	21.32	7,280,000
FFIYNREYNDALKLG	1861.936	15	931.9765	14.91	6,790,000
FNILVR	760.4595	6	761.4681	12.27	4,550,000
FNLLVR	760.4595	6	761.4681	12.27	4,550,000
FIPEYLS	867.4378	7	868.4468	15.03	3,610,000
GALFLQDNLVK	1216.682	11	1217.689	14.83	3,320,000

## Data Availability

The original contributions presented in this study are included in the article/supplementary material. Further inquiries can be directed to the corresponding author.

## Supplementary Materials

The following supporting information can be downloaded at: https://www.mdpi.com/article/10.3390/nu17091596/s1, Table S1. Experimental values and coded levels of the independent variables. Table S2. The treatment of animals. Table S3. The primers used in qRT-PCR analysis. Table S4. Variance analysis results of the influence of enzymolysis parameters of the SPP. Figure S1. Interaction between temperature and pH (A), temperature and enzyme dosage (B), pH and enzyme dosage (C), pH and time (D), temperature and time (E), enzyme dosage and time (F). Figure S2. Molecular weight distribution of SPP.
