# Supplementary material for: Anti-Hyperuricemic and Nephroprotective Effects of Hydrolysate Derived from Silkworm Pupae (Bombyx mori): In Vitro and In Vivo Study
Source: Nutrients. 2025 May 6;17(9):1596. doi: 10.3390/nu17091596 (PMC12073332; doi:10.3390/nu17091596)
Supplement: Supplementary file 1 [file nutrients-17-01596-s001.zip › nutrients-3584079-supplementary.pdf]

Supplementary data

Figure captions:

Figure S1. Interaction between temperature and pH (A), temperature and enzyme dosage (B), pH and enzyme dosage (C), pH and time (D), temperature and time (E), enzyme dosage and time (F).

Figure S2. Molecular weight distribution of SPP

Figure S1

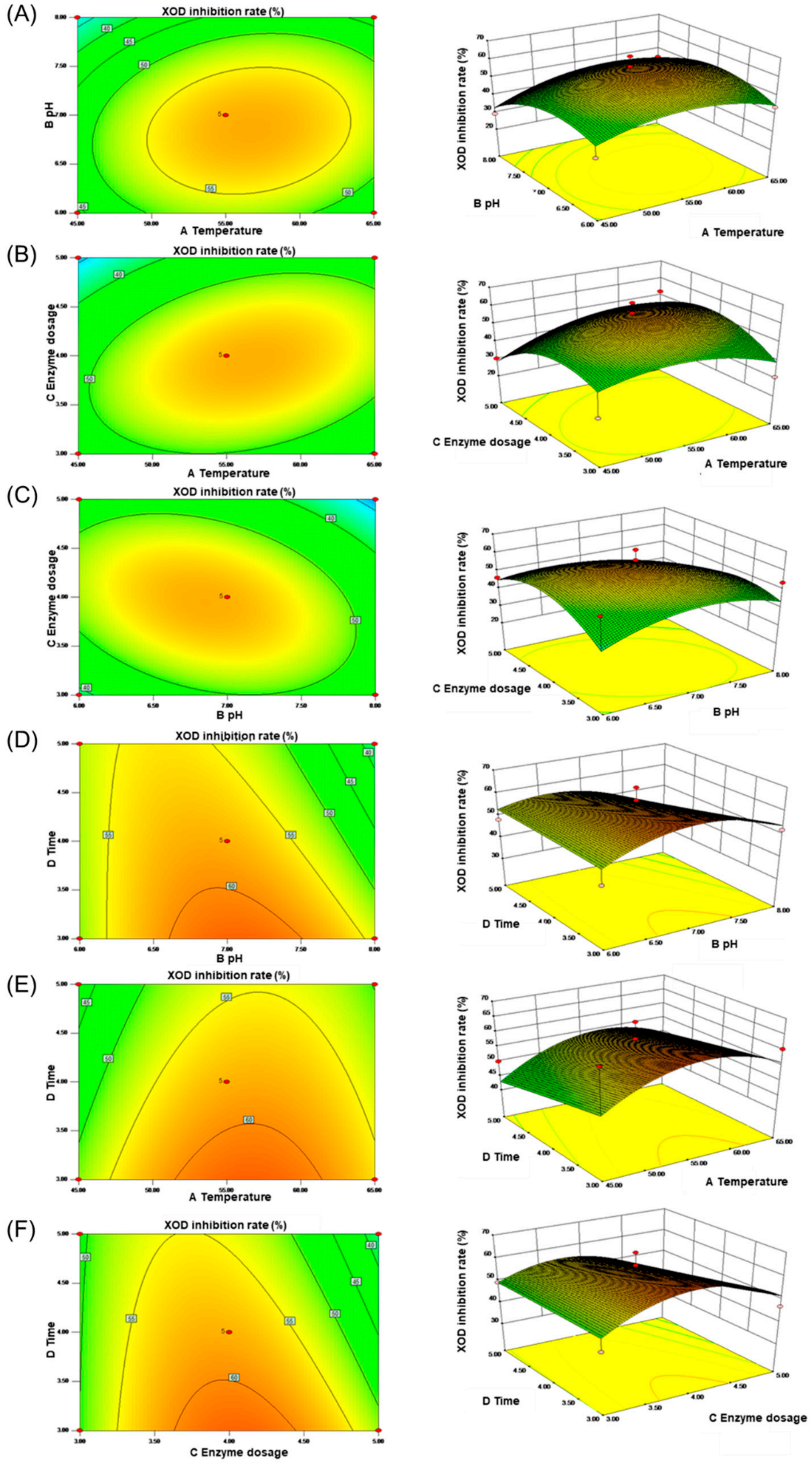

(A)
XOD inhibition rate (%)
B pH
A Temperature
XOD inhibition rate (%)
(B)
C Enzyme dosage
A Temperature
(C)
C Enzyme dosage
B pH
(D)
D Time
B pH
(E)
D Time
A Temperature
(F)
D Time
C Enzyme dosage

Figure S2

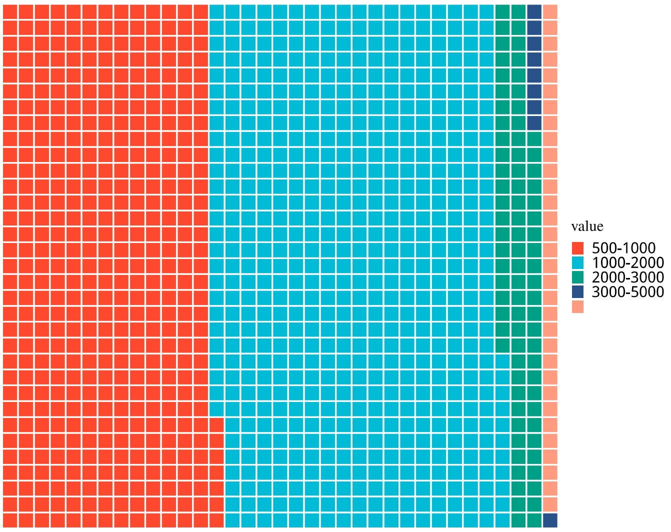

Tables

Table S1. Experimental values and coded levels of the independent variables

Level	Factor			
	A Temperature (℃)	B pH	C Enzyme dosage (kU/g)	D Time (h)
-1	45	6	3	3
0	55	7	4	4
1	65	8	5	5

Table S2. The treatment of animals.

Group	Time	
	0-7 days	8-28 days
NC	0.5% CMC-Na	Saline
MC	240 mg/kg PO+120 mg/kg hypoxanthine+0.5% CMC-Na	Saline
FEB		8 mg/kg febuxostat
SPP-L		0.25 g/kg SPP
SPP-M		0.50 g/kg SPP
SPP-H		1.0 g/kg SPP

Table S3. The primers used in qRT-PCR analysis

Gene	Forward primer	Reverse prime
OAT1	CAGCTGATCCAGGTCACCATG	CTGAGGTTGGCATTAGCAGGT
ABCG2	CCACGACTGGTTTGGACTCA	CTTAAAGATGGAATACCGAGGCT
β-actin	AAATGTGGCTGAGGACTTTGTAC	GGACTTCCTGTAACCACTTATTTCA

Table S4. Variance analysis results of the influence of enzymolysis parameters of the SPP.

Source	Sum of Squares	df	Mean Square	F Value	p-value Prob>F	
Model	291.31	14	20.81	5.78	0.0011	significant
A	59.00	1	59.00	16.40	0.0012	
B	2.54	1	2.54	0.71	0.4150	
C	0.41	1	0.41	0.11	0.7416	
D	5.50	1	5.50	1.53	0.2365	
AB	6.83	1	6.83	1.90	0.1898	
AC	0.71	1	0.71	0.20	0.6643	
AD	2.02	1	2.02	0.56	0.4664	
BC	6.69	1	6.69	1.86	0.1943	
BD	0.29	1	0.29	0.081	0.7795	
CD	5.12	1	5.12	1.42	0.2528	
A	63.32	1	63.32	17.60	0.0009	
B	168.01	1	168.01	46.69	<0.0001	
C	17.02	1	17.02	4.73	0.0473	
D	23.24	1	23.24	6.46	0.0235	
Residual	50.38	14	50.38			
Lack of Fit	44.92	10	4.49	3.29	0.1311	not significant
Pure Error	5.46	4	1.36			
Cor Total	341.68	28				
